# Intravenous Immunoglobulin for Hypogammaglobulinemia after Lung Transplantation: A Randomized Crossover Trial

**DOI:** 10.1371/journal.pone.0103908

**Published:** 2014-08-04

**Authors:** David J. Lederer, Nisha Philip, Debbie Rybak, Selim M. Arcasoy, Steven M. Kawut

**Affiliations:** 1 Department of Medicine, College of Physicians and Surgeons, Columbia University, New York City, New York, United States of America; 2 Department of Epidemiology, Mailman School of Public Health, Columbia University, New York City, New York, United States of America; 3 Department of Medicine, Perelman School of Medicine, University of Pennsylvania, Philadelphia, Pennsylvania, United States of America; 4 Center for Clinical Epidemiology and Biostatistics, Perelman School of Medicine, University of Pennsylvania, Philadelphia, Pennsylvania, United States of America; University of California Los Angeles, United States of America

## Abstract

**Background:**

We aimed to determine the effects of treatment with intravenous immunoglobulin on bacterial infections in patients with hypogammaglobulinemia (HGG) after lung transplantation.

**Methods:**

We performed a randomized, double-blind, placebo-controlled two-period crossover trial of immune globulin intravenous (IVIG), 10% Purified (Gamunex, Bayer, Elkhart, IN) monthly in eleven adults who had undergone lung transplantation more than three months previously. We randomized study participants to three doses of IVIG (or 0.1% albumin solution (placebo)) given four weeks apart followed by a twelve week washout and then three doses of placebo (or IVIG). The primary outcome was the number of bacterial infections within each treatment period.

**Results:**

IVIG had no effect on the number of bacterial infections during the treatment period (3 during IVIG and 1 during placebo; odds ratio 3.5, 95% confidence interval 0.4 to 27.6, p = 0.24). There were no effects on other infections, use of antibiotics, or lung function. IVIG significantly increased trough IgG levels at all time points (least square means, 765.3 mg/dl during IVIG and 486.3 mg/dl during placebo, p<0.001). Four serious adverse events (resulting in hospitalization) occurred during the treatment periods (3 during active treatment and 1 during the placebo period, p = 0.37). Chills, flushing, and nausea occurred during one infusion of IVIG.

**Conclusions:**

Treatment with IVIG did not reduce the short-term risk of bacterial infection in patients with HGG after lung transplantation. The clinical efficacy of immunoglobulin supplementation in HGG related to lung transplantation over the long term or with recurrent infections is unknown.

**Trial Registration:**

Clinicaltrials.gov NCT00115778

## Introduction

Potent immunosuppressive regimens, consisting of a calcineurin inhibitor, an anti-metabolite, and corticosteroids, predominantly target cell-mediated immunity to prevent lung allograft rejection after lung transplantation. Not surprisingly, lung transplant recipients suffer from an increased risk of infection by pathogens such as *Pseudomonas aeruginosa, Staphylococcus aureus,* and cytomegalovirus despite intensive antimicrobial prophylaxis [1], [2], [3], [4], [5].

Immunosuppressive therapy after solid organ transplantation may also contribute to humoral immunodeficiency due to hypogammaglobulinemia (HGG). [6], [7], [8], [9] A recent meta-analysis suggested that severe HGG after solid organ transplantation is associated with an increased risk of early infection and all-cause mortality. [10] In one study of lung transplant recipients, HGG was identified in 70% of lung transplant recipients, of whom 50% had very low immunoglobulin G (IgG) levels (<400 mg/dL). [9] Bacterial, fungal, and viral infections were significantly more common and survival significantly worse among those with HGG. We previously found that 58% of lung transplant recipients had mild incident HGG and 15% had severe HGG, with most episodes occurring within the first year of transplantation. [7] In that study, use of mycophenolate mofetil was an independent risk factor for HGG. We have also shown that the presence of HGG is associated with an increased risk of pneumonia, supporting the clinical importance of HGG in our lung transplant recipients. [8] Moreover, HGG has been reported in recipients of other solid organ transplants, such as heart and kidney, with significant clinical implications [11], [12], [13].

Intravenous immunoglobulin (IVIG) therapy is the current standard of care for patients with primary and certain secondary immunodeficiency states. Presently, IVIG is FDA-approved for treatment of primary humoral immunodeficiency, Kawasaki syndrome, B-cell chronic lymphocytic leukemia, and bone marrow transplant recipients with recurrent infections, pediatric HIV infection, and idiopathic thrombocytopenic purpura. It is well-established that augmentation of immunoglobulin levels in these immunodeficiency states results in decreases in bacterial infections [14].

IVIG therapy could significantly decrease the incidence and/or severity of infections in lung transplant recipients with HGG, however the use of IVIG in HGG after solid organ transplantation has not been well-studied. Despite the potential benefits, IVIG is relatively difficult to administer (requiring monthly intravenous infusion), has potential adverse reactions, and is very expensive. We performed a pilot phase II clinical trial to determine the efficacy and safety of immunoglobulin supplementation for HGG after lung transplantation.

## Materials and Methods

The protocol for this trial and supporting CONSORT checklist are available as supporting information; see Checklist S1 and Protocol S1.

### Ethics Statement

The protocol was approved by the Columbia University Medical Center (CUMC) Institutional Review Board and the medical monitor.

### Study Design

This was a single-center, randomized double-blind, placebo-controlled, two-period crossover study to determine the efficacy and safety of IVIG in patients with HGG after lung transplantation. The original protocol called for the recruitment of 10 subjects; we enrolled one additional subject after one dropped out. The first subject was randomized in April 2006 and the last subject by July 2008.

The manuscript was written by the authors, and the decision to submit the manuscript for publication was made solely by the authors. This manuscript was sent to the sponsor before submission for publication, however the sponsor had no right to delay or require any revisions to this manuscript. The sponsor did not participate in data analysis or drafting of the manuscript.

### Study Participants

We recruited adult lung transplant recipients at Columbia University Medical Center (CUMC) who were at least 3 months out from lung transplantation and had HGG (IgG levels <500 mg/dL) on two assessments at least one month apart within three months. Study subjects had to be on a stable medical regimen for at least one month before enrollment. We excluded those with acute rejection (defined histologically) or active infection within one month before enrollment, a contraindication to IVIG (acute renal failure, severe selective IgA deficiency, known hypersensitivity to IVIG), pregnancy, or a history of a thrombotic event within three months. The initial protocol called for IgG levels <400 mg/dL, which was increased to <500 mg/dL due to slow enrollment. All participants provided written informed consent. This study was registered at clinicaltrials.gov before the initiation of enrollment (NCT00115778) (http://clinicaltrials.gov/show/NCT00115778).

### Study Procedures

This was a crossover study with two 12 week treatment periods (separated by a 12 week washout period) comparing immune globulin intravenous (Human), 10% Caprylate/Chromatolgraphy Purified (Gamunex) (Bayer, Elkhart, IN) (“IVIG”) with 0.1% albumin (“placebo”). The *in vivo* half-life of this form of IVIG is approximately 35 days. The dose of IVIG in this study was 400 mg/kg every 4 weeks, which is a widely used dosage in clinical practice. The initial infusion rate was 0.01 mL/kg/min, titrated to a maximum infusion rate of 0.08 mL/kg/min. Placebo was 0.1% albumin in an equal volume, prepared by the Columbia Research Pharmacy. IVIG and placebo infusion bags had identical color and appearance. All participants were pre-medicated with acetaminophen 650 mg po and diphenhydramine 25 mg po before every study drug infusion.

The Research Pharmacy at Columbia University randomly assigned the treatment order. Investigators, study personnel, and study participants were blinded to the order and identity of the treatments. IGG levels were not performed during the study; samples were banked and then run after the conclusion of the study. Study drug was prepared by an unblinded research pharmacist and delivered to the infusion suite at our medical center where each dose of study drug was administered.

Study participants were evaluated at baseline and at Week 4, 8, 12, 24, 28, 32, and 36. The “Period 1” study drug was administered at baseline and at Week 4 and 8 study visits. The “Period 2” study drug was administered at Week 24, 28, and 32. There was a twelve week washout period between the Period 1 and Period 2. Phlebotomy was performed on the morning of each study visit, before infusion of the study drug. Participants maintained a diary of any new medications or symptoms during the study period.

### Outcome Assessments

The primary outcome was the number of bacterial infections during the treatment period. Secondary outcomes included viral, fungal and all non-bacterial infections, hospital admissions, antimicrobial use, serious bacterial infections, trough IgG levels, acute rejection, spirometry, and mortality in each period. The definitions of infectious end points were drawn from an FDA draft document. [15] The necessary criteria were collected prospectively and all infectious events were determined with blinding to the treatment period.

### Statistical Analysis

Generalized estimating equations with a compound symmetry covariance structure and logit link were used to estimate odds ratios. Linear mixed effects modeling with an autoregressive covariance structure were used to assess differences in lung function and IgG levels between treatment arms. Models included fixed effects for drug and period. Subject was included as a random effect. Least squares means and 95% confidence intervals for continuous outcomes are reported. Paired sample analysis was performed secondarily.

In order to detect a reduction in the mean number of bacterial infections/patient by one standard deviation over three months with 80% power at an α level of 0.05, we estimated that we would need 10 subjects. This effect estimate was similar to the difference between patients with and without HGG in prior studies. [9] The crossover design of the trial, in which each subject serves as his or her own control, allows sufficient power for the detection of a clinically significant effect of IVIG with only a small number of patients and is the major strength of this design. For example, a sample size of 10 patients in a crossover trial has more power to detect differences than double the sample size using a parallel group design (i.e., randomizing 10 patients to IVIG and 10 patients to placebo). As we did not have multiple bacterial infections per patient (see below), the power of the study was less than anticipated.

The primary analysis proceeded according to the intent-to-treat principle. All randomized participants were analyzed in their originally assigned group whether or not they discontinued study drug. P values<0.05 were considered significant. Analyses were performed using SAS 9.2 (SAS Institute, Cary, NC). There were no interim analyses or stopping rules planned *a priori* for the trial.

## Results

**Figure 1:**
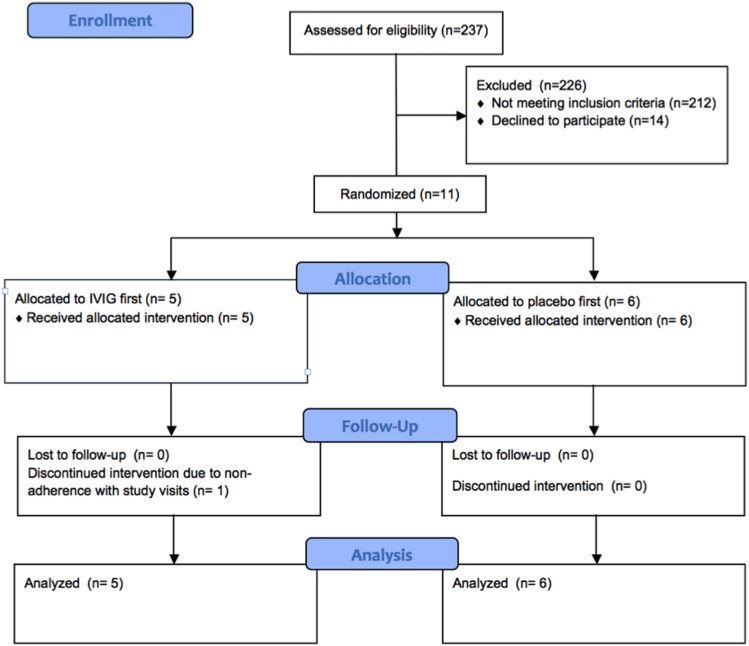
Study flow.

We screened 237 lung transplant recipients between January 2005 and July 2008 (Figure 1). Eleven subjects were eligible and randomized, and 10 completed all study assessments. One subject discontinued the interventions because of inability to comply with the schedule of study visits.

Participant characteristics are shown in Table 1. The mean age was 55 years (range 28 to 66 years) and six (55%) were male. Most were non-Hispanic white. Six underwent lung transplantation for COPD and ten had undergone bilateral transplantation. The median time from transplantation to enrollment was 187 days (interquartile range 153 to 660 days). Five study participants (45%) were taking valganciclovir for prophylaxis against cytomegalovirus. The median baseline IgG level for the study sample at randomization was 487.5 g/dl (IQR 423.5 to 513.8 g/dl), showing the variability over time in IgG levels (as all patients had IgG levels <500 g/dl on two assessments at least one month apart within three months before enrollment).

**Table 1:** Participant characteristics.

Characteristic	Value
No.	11
Age	60 (28 to 66)
Male	6
Race/ethnicity	
Non-Hispanic white	10
Hispanic white	1
Height, cm	168 (140 to 183)
Weight, kg	68 (52 to 88)
Pre-transplant diagnosis	
Chronic obstructive pulmonary disease	6
Interstitial lung disease	2
Cystic fibrosis	1
Pulmonary arterial hypertension	2
Transplant procedure	
Bilateral	10
Single	1
Days since transplantation	187 (119 to 1330)
Serum creatinine, mg/dl	1.3 (0.9 to 1.8)
Immunosuppressive medications	
Tacrolimus	9
Cyclosporine	2
Azathioprine	5
Mycophenolate mofetil	6
Prednisone	11
Forced vital capacity, % predicted	92 (60 to 112)
Forced expiratory volume in 1 sec, % predicted	109 (65 to 121)
Forced expiratory volume in 1 sec/Forced vital capacity ratio, %	88 (72 to 100)
Forced expiratory flow 25–75, % predicted	128 (50 to 270)

Data are median (range) and frequency.

Subjects received the study treatments in the assigned order. One participant experienced chills, flushing, and nausea during infusion of IVIG. No other infusion-related adverse events occurred. Study drug was discontinued in one subject because of inability to comply with study visits. There were no period or carryover effects.

**Figure 2:**
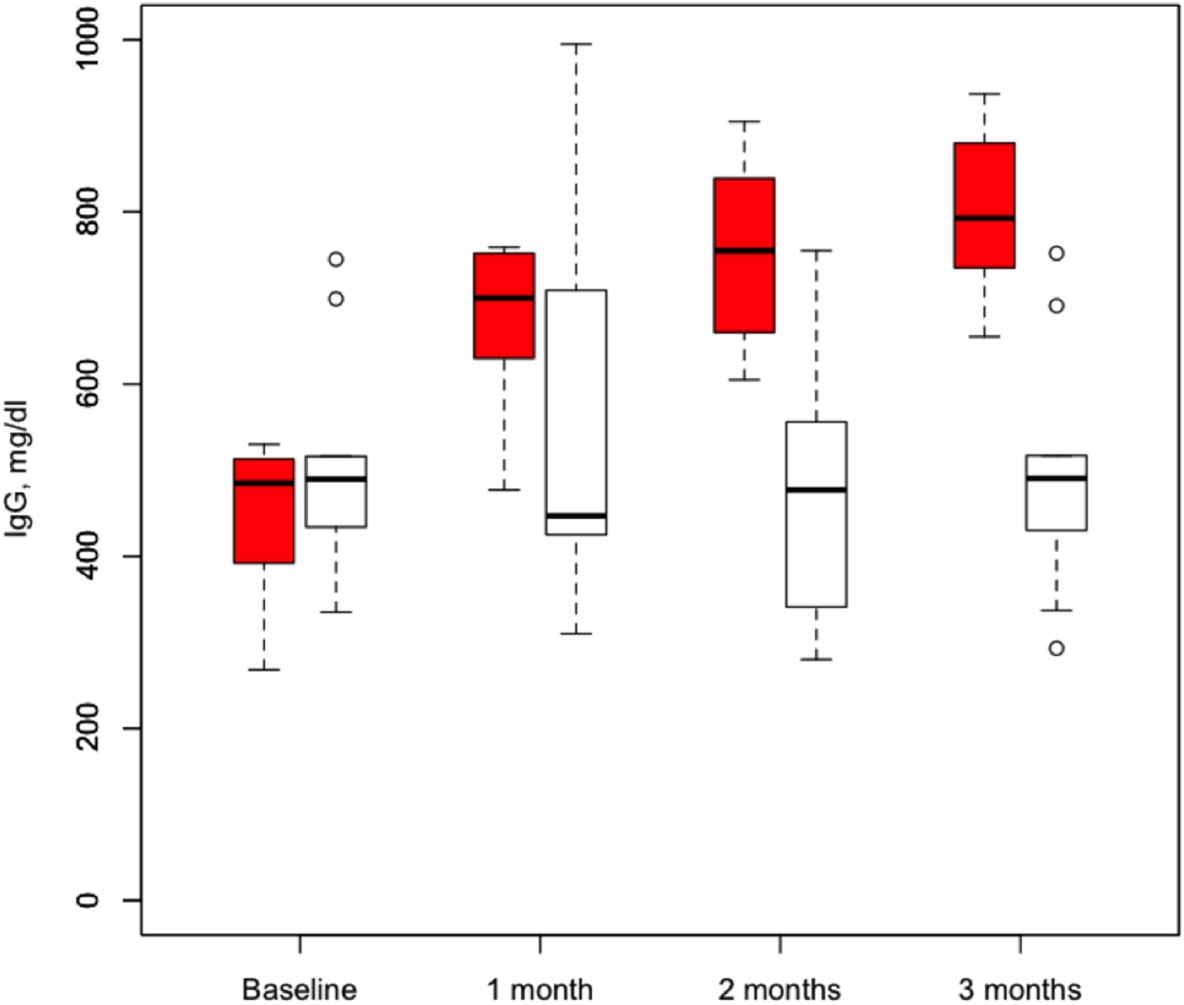
Box (interquartile range) and whisker plots of serum IgG levels during the IVIG period (red) and placebo period (white).

During the IVIG period, there were three bacterial infections (in three patients) compared to the placebo period, when there was one bacterial infection (in one patient) (odds ratio (OR) 3.5, 95% CI 0.4 to 27.6, p = 0.24; Table 2). Paired sample analyses showed similar results (data not shown). Similarly, there were non-significant increases in overall infections (IVIG-7 vs. placebo-3, OR 2.7, 95% CI 0.95 to 7.6, p = 0.06), antibiotic initiation (IVIG-9 vs. placebo-8, OR 1.4, 9% CI 0.3 to 6.0, p = 0.61), and hospitalization (IVIG-3 vs. placebo-1, OR 3.5, 95% CI 0.2 to 51.2, p = 0.37; Table 2) during the IVIG period compared to the placebo period. IVIG had no significant effect on lung function over three months (Table 3). As expected, the administration of IVIG significantly increased IgG levels throughout the treatment period compared to placebo (Table 3; Figure 2).

**Table 2:** Outcomes.

	No. ofeventsoccurringduringIVIG treatment period	No. ofeventsoccurringduringplacebo treatmentperiod	Oddsratio(IVIG vs. placebo)	95% confidence interval	P value
Bacterial infection	3	1	3.5	0.4 to 27.6	0.24
Viral infection	2	2	0.8	0.1 to 5.9	0.87
Fungal infection	2	0	–	–	
Any infection	7	3	2.7	0.95 to 7.6	0.06
Positive culture	4	2	2.0	0.4 to 9.3	0.37
Antibiotic initiation	9	8	1.4	0.3 to 6.0	0.61
Bronchoscopy	7	6	1.3	0.7 to 2.4	0.47
Hospitalization	3	1	3.5	0.2 to 51.2	0.37
Acute rejection	0	0	–	–	
Lymphocytic bronchiolitis	0	0	–	–	

**Table 3:** Least square means (95% confidence interval) for spirometry value and serum IgG levels after IVIG and placebo.

	IVIG	Placebo	P value
Forced vital capacity, % predicted	88.8 (86.6–91.1)	89.7 (87.4–92.0)	0.51
Forced expiratory volume in 1 sec, % predicted	95.3 (92.2–98.3)	95.6 (92.5–98.7)	0.84
Forced expiratory volume in 1 sec/forced vital capacity ratio, %	86.9 (84.6–89.1)	85.3 (83.0–87.6)	0.14
Forced expiratory flow 25–75, % predicted	125.0 (114.6–135.4)	119.0 (108.5–129.5)	0.14
IgG, mg/dl	765.3 (720.1–810.6)	486.3 (441.0–531.5)	<0.001

Table 4 shows the adverse events during the IVIG and placebo periods. Five serious adverse- events occurred during the study, and four during a treatment period. Three (pancreatitis, vitreous hemorrhage, and E. coli pneumonia) occurred during the IVIG period and one (hospital admission for thymoglobulin infusion) during the placebo period. There was a hospitalization for a fall during the washout period. The most common adverse events were bronchoscopy (in >50% of participants in both periods). Cough and neck stiffness were more common during IVIG periods.

**Table 4:** Adverse events.

Event	IVIG	Placebo	Washout
Any AE	11 (100%)	11 (100%)	11 (100%)
Serious adverse event			
Any SAE	3 (27%)	1 (9%)	1 (9%)
Infusion-related AEs*			
Any infusion-related AE	1 (9%)	0 (0%)	–
Chills	1 (9%)	0 (0%)	–
Flushing	1 (9%)	0 (0%)	–
Nausea	1 (9%)	0 (0%)	–
Infectious			
Fever	2 (18%)	0 (0%)	2 (18%)
Night sweats	0 (0%)	2 (18%)	0 (0%)
Bronchoscopy	7 (64%)	6 (54%)	7 (64%)
Pulmonary			
Dyspnea	1 (9%)	1 (9%)	2 (18%)
Cough	2 (18%)	1 (9%)	1 (9%)
Sputum production	1 (9%)	1 (9%)	1 (9%)
Cardiac			
Palpitations	1 (9%)	1 (9%)	1 (9%)
Pedal edema	0 (0%)	3 (27%)	0 (0%)
Neurological			
Headache	2 (18%)	3 (27%)	0 (0%)
Stiff neck	2 (18%)	0 (0%)	1 (9%)
Genitourinary			
Urinary frequency	1 (9%)	2 (18%)	0 (0%)
Pancreatitis	1 (9%)	0 (0%)	0 (0%)
Gastrointestinal			
Diarrhea	0 (0%)	2 (18%)	1 (9%)
Abdominal discomfort	1 (9%)	2 (18%)	0 (0%)
Heartburn/GER	1 (9%)	1 (9%)	0 (0%)
Other			
Musculoskeletal pain	2 (18%)	2 (18%)	0 (0%)
Acute kidney injury	1 (9%)	0 (0%)	0 (0%)
Vitreous hemorrhage	1 (9%)	0 (0%)	0 (0%)

*AEs with more than 1 occurrence during the study (except for infusion related AEs, acute kidney injury, pancreatitis, and vitreous hemorrhage).

## Discussion

To our knowledge, this is the first randomized, double-blind, placebo-controlled clinical trial of IVIG for HGG after lung transplantation. In a crossover design, we did not find a significant impact of IVIG on bacterial infections or other infectious episodes, despite observing expected increases in IgG levels following IVIG administration. Adverse events were mild and common during both IVIG and placebo periods.

Previous studies have shown associations between the presence of HGG after lung transplant and worse outcomes. We previously demonstrated that lung transplant recipients with severe HGG (IgG<400 mg/dl) had a higher cumulative incidence of pneumonia (63%) compared to that of recipients without HGG (18%), (p = 0.01), but there was no significant increase in the risk of CMV disease. [8] Those with severe HGG also had an increased risk of death. The incidence of severe HGG was about 15% in our prior studies, [7], [8] however other investigators have demonstrated even higher prevalences of HGG after lung transplantation. [9] A recent study in pediatric lung transplant recipients found an association between HGG and the risk of infections and hospitalization. [16] More recently, lower post-transplant IgG levels were associated with an increased risk of BOS [6].

IVIG is the current standard in clinical practice for replacement therapy of patients with primary immunodeficiency states. [17] Currently, IVIG is FDA approved for treatment of primary humoral immunodeficiency, Kawasaki syndrome, chronic lymphocytic leukemia and bone marrow transplant recipients with recurrent infections and idiopathic thrombocytopenic purpura. While supplementation using IVIG in HGG after solid organ transplantation would be presumed to be effective, there are several reasons why it may not be. Organ transplant recipients receive a medical regimen that not only reduces B cell function (and antibody production), but also T cell function. HGG might be an epiphenomenon of reduced T cell function and merely replacing antibodies might not improve defenses against infection.

To our knowledge, there is only one other RCT of immunoglobulin replacement for HGG in thoracic organ transplantation. Yamani et al. studied the use of Cytogam in patients with IgG levels between 350 and 500 mg/dl in a randomized, double-blind placebo controlled trial with 13 patients randomized to Cytogam and 10 patients randomized to placebo (5% dextrose). [18] An average of only 1.4 doses were administered per patient, without a standard regimen. The investigators found a decrease in the number of episodes of CMV disease, but no other differences between the groups. These results may not be generalizable as this study 1) used Cytogam in patients who received valganciclovir for CMV prophylaxis for only 4 weeks after transplantation, 2) on average only administered 1–2 doses of study drug for the entire study period, 3) only showed an effect on CMV infection (defined as a positive CMV PCR with fever, malaise, leukopenia, or other evidence of organ disease), and 4) demonstrated no effect on bacterial infections. These data may be difficult to extrapolate to the chronic use of IVIG in lung transplant patients with HGG receiving more prolonged CMV prophylaxis.

Our study had several limitations. While the crossover design provided more power than we would have had with >20 subjects studied in a parallel design, there were few outcomes and our findings could very well be attributable to inadequate power. For the crossover design to be valid, we needed to ensure clinical stability over the time of both treatment periods, necessarily limiting the duration of each. Longer studies with greater numbers of patients would be necessary to detect smaller differences in outcomes. The primary statistical analysis may be anti-conservative in small samples, however no differences in the primary end point were seen and paired sample analyses were similar. We recruited patients with low IgG levels documented twice over a short period of time. Even so, some of the patients in the study had IgG levels >500 mg/dl at the time of randomization and during the placebo period. This was not attributable to a carryover effect (i.e., the effect of IVIG treatment during the first period continuing into the second period), but likely reflects the variable nature of IgG levels over time. While reflecting the “real world” application of IVIG (i.e., some patients clinically diagnosed with HGG based on two IgG levels in whom IVIG might be prescribed will go on to have IgG levels which are higher), it is possible that IgG levels >500 mg/dl during the placebo period could have led to bias to the null, even though the study subjects had significantly higher levels of IgG at every time point during treatment with IVIG. Finally, one participant dropped out early in the first period, which could have reduced power as well.

In summary, monthly IVIG therapy did not reduce the incidence of bacterial or other infections compared to placebo over three months in stable adult lung transplant recipients with HGG. Our results do not support the routine clinical use of IVIG in patients with HGG after lung transplant. However, it is unknown whether IVIG may be effective for lung transplant recipients with HGG and repeated or refractory bacterial, fungal, or viral infections.

## Supporting Information

Checklist S1 **CONSORT checklist.**
(DOC)

Protocol S1 **Study Protocol.**
(DOC)
