# Supplementary material for: Intravenous Immunoglobulin for Hypogammaglobulinemia after Lung Transplantation: A Randomized Crossover Trial
Source: PLoS One. 2014 Aug 4;9(8):e103908. doi: 10.1371/journal.pone.0103908 (PMC4121238; doi:10.1371/journal.pone.0103908)
Supplement: Protocol S1 — Study Protocol. (DOC) [file pone.0103908.s002.doc]

1. Study Purpose and Rationale

Potent immunosuppressive regimens, consisting of a calcineurin inhibitor, an anti-metabolite, and corticosteroids, predominantly target cell-mediated immunity to prevent lung allograft rejection. An increased risk of infection despite intensive antimicrobial prophylaxis is a well-recognized complication of lung transplantation. The risk of infection is higher in lung transplant recipients than in other solid organ recipients due to 1) higher levels of pharmacologic immunosuppression for longer periods of time, 2) frequent episodes of acute rejection requiring further intensification of immunosuppressive therapy, 3) the development of chronic rejection (bronchiolitis obliterans) and subsequent bronchiectasis, and 4) continuous exposure of the lungs to the upper respiratory and gastrointestinal tracts, the outside environment, and the native lung (in single lung transplantation). It is therefore not surprising that infectious complications occur in lung transplant recipients twice as frequently as in heart transplant recipients. Bacteria and CMV account for most of these infections with Pseudomonas aeruginosa and Staphylococcus aureus being the most commonly isolated bacterial agents.

Recent evidence suggests that immunosuppressive therapy after solid organ transplantation may lead to humoral immunodeficiency due to hypogammaglobulinemia (HGG). Although the T-cell effects of immunosuppressive medicines may impair B-cell function, agents such as mycophenolate mofetil may also affect B cells directly. In a recent study of lung transplant recipients, HGG was identified in 70% of patients, of whom 50% had very low immunoglobulin G (IgG) levels (<400 mg/dL). Bacterial, fungal, and viral infections were significantly more common and survival significantly worse in the IgG-deficient group. We have also documented the prevalence and clinical importance of HGG in our lung transplant recipients. Moreover, HGG has been reported in recipients of other solid organ transplants, such as heart and kidney, with significant clinical implications.

Bacterial and viral colonization of the lungs occurs in patients with HGG. In a recent study, Haemophilus influenzae was the most common bacteria isolated from the lungs of patients with primary HGG. Viruses, including adenovirus, rhinovirus and CMV, was also commonly found in these patients. Prevention of this microbiological colonization in patients with HGG after lung transplantation could result in considerable reduction in infection risk and improved outcomes.

Intravenous immunoglobulin (IVIG) therapy is the current standard in clinical practice for replacement therapy in patients with primary and certain secondary immunodeficiency states. Presently, IVIG is FDA-approved for treatment of primary humoral immunodeficiency, Kawasaki syndrome, chronic lymphocytic leukemia and bone marrow transplant recipients with recurrent infections, and idiopathic thrombocytopenic purpura. It is well-established that augmentation of immunoglobulin levels in these diseases results in decreases in bacterial infections.

In lung transplant recipients with HGG, IVIG therapy offers the potential to significantly decrease the incidence and/or severity of infections, thereby reducing morbidity and potentially mortality. Additionally, with IVIG replacement, infection prophylaxis measures can potentially be de-intensified and/or shortened, resulting in less frequent drug toxicity, such as leukopenia, renal insufficiency, and allergic reactions. As CMV pneumonitis predisposes to chronic rejection, the proven ability of IVIG to prevent CMV infection might translate into a reduction in the incidence of this “Achilles heel” of lung transplantation.

Despite these potential benefits, IVIG is relatively difficult to administer (requiring monthly intravenous infusion), has potential adverse reactions, and is very expensive (> $12,000/year/patient). To date, randomized controlled trials in a solid organ transplant population have not been published. While the transplant research community is aggressively pursuing immunotolerance to enhance graft and patient survival, this concept is at least several years away for clinical transplantation. We therefore propose a pilot study of an established therapy which could be implemented today, given evidence of its safety and efficacy. Considering the suboptimal survival rates and the impact of infections in lung transplant recipients, HGG may be a treatable vulnerability in the complicated post-operative course after lung transplantation. With its ability to augment immunity to pathogenic organisms without increasing the risk of rejection, IVIG therapy could be a significant advance in post-transplant management of lung and other solid organ transplant recipients.

2. Study Design and Statistical Procedures

We will perform a Phase II double-blind, two-period, two treatment, placebo-controlled crossover trial of IVIG therapy. A cross-over design is attractive for its reduced variance and subsequent reduced sample size requirements due to within-subject comparisons. As underlying clinical stability is an essential assumption of this study design, we will select patients who are farther from transplant and have shown stability in their immunosuppressive regimens. Previous crossover trials of IVIG have established a 3 month wash-out period as sufficient to avoid a carry-over effect.

We will perform the analysis by intention-to-treat. For Specific Aim 1, the number of bacterial infections per patient will be compared between the treatment and control periods. In addition, the number of non-bacterial and total infections will be compared between the periods. If the continuous data do not meet the assumption of normality, they will be transformed, as needed. For Specific Aim 2, the number of bacteria either bound to or ingested by 100 macrophages from each subject during the treatment and control periods will be compared.

The main method of analysis will be the linear mixed-effects model. The outcome variables will be the number of bacterial infections (Aim 1) and the phagocytosis indices and the extent of bacterial killing (Aim 2). The predictor of interest will be the treatment (IVIG or placebo). Period number will be included as a covariate. The correlation between outcomes from the same subject will be modeled using a random subject-specific intercept term. The interactions between treatment and presence of chronic rejection will be examined. Interactions between period and treatment will also be examined. Analysis of time-to-infection in each period will also be performed using proportional hazards modeling adapted for the crossover study design. The variables included in the final models will be chosen on the basis of the statistical significance of estimates, the effect of their inclusion on other factors in the model, and on scientific plausibility. Analyses will also include trough immunoglobulin levels. Tests for trends over time will be performed using the baseline measurements of IgG levels. Binary outcomes will be analyzed using appropriate methods.

Sample Size

The Lung Transplantation Center currently cares for approximately 100 patients who are beyond 3 months of their transplantation procedure, and we perform approximately 25 transplants/year. We anticipate 14% of these patients to have severe HGG. Over a recruitment period of one year, we therefore anticipate 3 new patients with severe HGG, in addition to at least 14 prevalent cases (100 post-transplant patients alive at start of recruitment x 14% severe HGG). Of these 17 patients, we expect more than 80% to meet inclusion criteria and less than 10% to refuse to participate. As lung transplant recipients have an uncommon and serious medical condition requiring multiple medications, procedures, and hospitalizations, they are particularly eager to participate in clinical trials focused on improving clinical outcomes. We have found similar refusal rates in other studies of patients undergoing lung transplantation.

We have based our power calculations for this protocol on the reduction in the number of bacterial pneumonias in lung transplant patients with severe HGG. Assuming an α level of .05 and a β level of .20, we will be able to detect a reduction in the mean number of bacterial pneumonias/patient by one standard deviation over three months with 10 subjects. This reduction in pneumonias in patients with severe HGG approximates the number of bacterial pneumonias in lung transplant patients with normal immunoglobulin levels, and is therefore a clinically significant difference. The crossover design of this trial, in which each patient serves as his or her own control, allows the detection of a clinically significant effect of IVIG with only a small number of patients. This detectable difference also represents a significant improvement in the number of bacteria bound or ingested by phagocytes for the in vitro assays.

If more patients refuse to participate or drop-out is higher than expected (20%, for example), we would still have enough eligible patients from which to recruit additional subjects. As we have performed our sample size analyses based only on the outcome of bacterial pneumonia, the mean number of total bacterial infections in patients with HGG during the placebo period will likely be higher, making our power estimates for our primary analysis quite conservative.

3. Study Procedures

Eligible patients will be recruited from the Lung Transplantation Program. After consent is obtained, subjects will then be assigned a study number. The subject will be evaluated in the Transplant Center on Week #0. Peripheral blood will be drawn by venipuncture for assessment of immunoglobulin levels and in vitro phagocytosis assays.

The Office of Clinical Trials Pharmacy will randomly assign treatment order. Investigators, study personnel, and patients will be blinded to the order and identity of the treatments.

The patient will then receive Treatment #1 (IVIG or placebo). The subject will be seen again in the Transplant Center on Weeks #4 and #8 for repeat administration of Treatment #1 and repeat assessment of immunoglobulin levels (which will be banked and assessed at the conclusion of the study period) . On Week #12, the subject will be assessed with in vitro assays and immunoglobulin levels. The subject will return on Week #24 for repeat laboratory assessment and initiation of Treatment #2. The subject will receive additional doses of Treatment #2 on Week #28 and Week #32 and have immunoglobulin levels drawn. On week #36 in vitro assays and immunoglobulin levels will be performed. Subjects will be treated for a total of 6 months divided in two treatment periods. Patients will receive acetaminophen 650 mg po and diphenhydramine 25 mg po before the study drug infusion.

Data Collection

**Data to assess the occurrence of endpoints will be collected from the routine clinical care of the subjects in this trial. The work-up of possible infections in lung transplant recipeints is standardized, comprising of a history and physical exam, and laboratory and radiographic studies. These studies will be obtained as dictated by usual clinical care and not for the purposes of this study. Assessment for these endpoints will not entail additional testing.**

Endpoints:

Primary endpoints

Specific Aim 1: Number of bacterial infections (pneumonia, tracheobronchitis, sinusitis, bacteremia, line infection, urinary tract infection, central nervous system infection, cellulitis). All infection endpoints will be defined using standard guidelines for the evaluation of anti-infective drug products from the Infectious Diseases Society of America and the FDA.

Specific Aim 2: Number of bacteria (Pseudomonas aeruginosa and Staphylococcus aureus) either bound to or ingested by 100 macrophages in vitro.

Secondary endpoints

Viral, fungal, and all other non-bacterial infections, based on previously defined guidelines. We will also assess the number, type and duration of hospital admissions during the study period. The use of prophylactic and therapeutic antibiotics will be recorded. Duration of infection in terms of number of days with symptoms and number of febrile days (days with temperature equal to or less than 38.50C) will be assessed.

Other secondary endpoints will include side effects attributable to IVIG, trough levels of IgG, acute rejection episodes, FEV1%, FVC%, incidence of bronchiolitis obliterans syndrome, and mortality. **All of these tests are obtained as part of routine clinical care of the lung transplant recipient.**

At each drug or placebo administration, patients will be questioned regarding potential endpoints in the interim since their last visit. Potential endpoints will be identified by any symptoms, antibiotic use, or contact with either the transplant team or outside physicians. Patients will be examined during each infusion and a specially-trained nurse will assess vital signs and side effects during infusions.

At the conclusion of the study period, the medical records from all potential endpoints will be reviewed by a consensus panel of three physicians who are blinded to the treatment assignment and subject identity. They will review the potential endpoints using our explicit guidelines.

Assessment of Phagocytic Function

Peripheral blood (20ccs) will be obtained by venipuncture; mononuclear cells will be isolated by density centrifugation and monocytes will be purified from this fraction by adherence to tissue culture plastic in the presence of 20% human serum. In our hands this results in a purity of >90% monocytes. Following an overnight incubation, cells will be replated onto glass coverslips and maintained in a 5% CO2 incubator at 37ºC for 5 days to allow for differentiation into macrophages. Standard phagocytosis assays will be performed in triplicate using 107 colonies of P. aeruginosa (strain PAO1), methicillin-sensitive S. aureus (ATCC 29213) or a virulent capsular type 2 strain (D39) of S. pneumoniae. These strains were chosen as representative of the majority of bacterial infections that have been documented in primary HGG and in our lung transplant patients with severe HGG. Phagocytosis assays will be performed in the presence of 50% autologous serum and in the absence or presence of 10 mg/ml supplemental pooled human IgG. In addition, whole blood bactericidal assays will be performed as described. Parallel assays will be performed in the presence of supplemental pooled human IgG (10 mg/ml), which will serve as a control for the optimal magnitude of IgG-dependent phagocytosis and killing. All phagocytosis and whole-blood bactericidal assays will be performed in the laboratory of Steven Greenberg, M.D.

4. Study Drug

IVIG is a pooled plasma product that was FDA-approved for use in primary immunodeficiency syndromes in 1981. IVIG is also widely used in secondary immunodeficiency states, such as HIV infection, bone marrow transplantation, leukemia, and other disorders. We will use immune globulin intravenous (Human), 10% Caprylate/Chromatolgraphy Purified (Gamunex) (Bayer, Elkhart, IN) for this protocol. This preparation was selected based on the lower incidence of renal dysfunction with this product. The plasma source is over 2000 paid human donors. The in vivo half-life is approximately 35 days. IgG constitutes 95-99% of the fractionated serum with only traces of IgA, IgM, IgD and IgE. The usual maintenance dose is 300-600 mg/kg every 3 to 4 weeks, which reduces the incidence and severity of infections in primary immunodeficiencies. The dose of IVIG in this study will be 400 mg/kg every 4 weeks, which is the most widely utilized dosage in clinical practice. The initial infusion rate will be 0.01 mL/kg minute which will be titrated to a maximum infusion rate of 0.08 mL/kg/min. IVIG therapy is generally well-tolerated, with a 5% incidence of side effects. These include fever, chills, headache, back pain, chest pain, malaise, myalgia, nausea, vomiting, and rash. Most of these reactions are mild and self-limited, and often can be alleviated by reducing the rate of infusion. Major adverse reactions occur very infrequently, including a hypersensitivity reaction with anaphylaxis (mainly in IgA-deficient patients, who will be excluded), aseptic meningitis and nephrotoxicity. The transmission of HIV or hepatitis B with currently available products has not been reported. Early reports of hepatitis C transmission with the use of IVIG have led to significant changes in the manufacturing process, resulting in no recent reports of seroconversion. Placebo will be 0.1% Albumin in an equal volume, prepared by the Columbia Research Pharmacy. All patients will be pre-medicated with acetaminophen 650 mg po and diphenhydramine 25 mg po before the study drug infusion.

The Research Pharmacy at Columbia University will randomly assign treatment order. Investigators, study personnel, and patients will be blinded to the order and identity of the treatments. The immunoglobulin/placebo will be prepared by an unblinded Research Pharmacist and delivered to the infusion suite. The infusion protocols will be identical between placebo and active drug. The study drug/placebo will be administered in the infusion suite of the Beverly & Arthur Shorin Transplant Center.

5. Study Questionnaires

N/A

6. Study Subjects

Study Population

The subjects for this protocol will be 10 lung transplant recipients who are patients at the Columbia University Medical Center (CUMC) Lung Transplantation Program who were transplanted more than 3 months prior to recruitment. As the majority of changes in immunosuppressive therapy occur in the first 3 months following lung transplantation, patients will receive a stable immunosuppressive regimen at the end of this period. Additionally, HGG is not expected to occur within the first 3 months after transplantation and is typically observed after 6 months. **All patients are currently screened by quantitative serum immunoglobulin (IgG, IgA and IgM) assay as per the clinical protocol of the Lung Transplant Protocol of Columbia University.** **This is a routine procedure in the clinical care of transplant patients at our center.** Patients who are identified to have severe HGG (as defined by serum IgG levels less than 400 mg/dL) will be eligible for the crossover study. Eligible patients will be identified by the Key Personnel who comprise the CUMC Lung Transplant Team. **As is common in the study of rare diseases, the clinicians who are caring for the patients will recruit and consent patients for the study. Patients will be assured that their participation or lack thereof will not affect their clinical care.**

Inclusion criteria:

Lung transplant recipients > 3 months after transplant surgery

IgG < 400 mg/dL on two assessments at least one month apart within three months

Stable medical regimen for one month before enrollment

Exclusion criteria:

Acute rejection episode as defined histologically by the Lung Rejection Study Group within one month before enrollment

Active infection within one month of enrollment

Contraindication to IVIG (Acute renal failure, severe selective IgA deficiency, known hypersensitivity to IVIG)

Pregnancy

Recent (less than 3 months) history of thrombotic events

7. Recruitment

The investigators on this study are the physicians who provide all medical care for the potentially eligible subjects (lung transplant recipients at Columbia). The investigators will approach potentially eligible patients.

8. Confidentiality of Study Data

We will have several mechanisms in place to maintain confidentiality. Patient names will not be abstracted and all of the data will be reported in aggregate. Each subject in all phases of the study will be assigned a unique study code number to be used on all data forms and samples. A list of patient names and code numbers will be maintained separately in locked file cabinets or on password protected computers at the recruiting centers and centrally by the PI. Only the investigators and project staff will have access to this information. No other personally identifiable information will be available. The sponsor may have access to data which have been de-identified except for the study code number.

9. Potential Risks

We will obtain repeated blood samples from each subject. There is a risk of bruising, hematoma, and infection after phlebotomy, which are possible but not considered serious adverse events. Fainting may occur which is unlikely, but considered a serious adverse event.

Administration of IVIG is a potential risk. IVIG therapy is widely used and has become the current standard in clinical practice for replacement therapy in patients with primary and certain secondary immunodeficiency states. Presently, IVIG is FDA-approved for treatment of primary humoral immunodeficiency, Kawasaki syndrome, chronic lymphocytic leukemia and bone marrow transplant recipients with recurrent infections, and idiopathic thrombocytopenic purpura. IVIG therapy is generally well-tolerated, with a 5% incidence of side effects. These include fever, chills, headache, back pain, chest pain, malaise, myalgia, nausea, vomiting, and rash. Most of these reactions are mild and self-limited, and often can be alleviated by reducing the rate of infusion. Major adverse reactions occur very infrequently, including a hypersensitivity reaction with anaphylaxis (mainly in IgA-deficient patients, who will be excluded from the protocol), aseptic meningitis and nephrotoxicity.

Because of the possibility (albeit small) of a major adverse effect, IVIG or placebo will be administered with manufacturer-recommended infusion rates and monitored in the infusion suit of the Lung Transplant Center, which has extensive experience in infusion of IVIG and other intravenous therapies. The transmission of HIV or hepatitis B with currently available products has not been reported.

We will institute strict procedures to minimize the risks to the participants in this study. Blood sampling will be performed by trained phlebotomists. IVIG has been studied extensively in both normals and in patients with primary and secondary HGG and after bone marrow transplantation and is FDA-approved. Although IVIG has not been studied in patients after solid organ transplantation, it is expected that the side effects of the study infusion will be similar to these other populations. We will have specially-trained infusion nurses supervise all IVIG and placebo infusions. The nurses will have standardized protocols for responding to side effects with alteration in infusion rates or cessation of infusions with severe adverse events. Subjects will be examined by a physician at each study visit. We will use Gamunex which has a lower risk of nephrotoxicity than other IVIG preparations.

10. Potential Benefits

There may be no direct benefits. The results from the study could be applied in the future to patients (including those in the study) who stand to benefit from the information. As future studies of HGG may emerge from this study, there may be significant benefit for patients with HGG (including those in this trial). As the study involves the risks of phlebotomy and administration of IVIG, and there is a potential for future benefit for both subjects in the study and for future patients, the risk/benefit ratio is favorable.

The knowledge to be gained from this study is potentially very important. The clinical impact of HGG and the effect of IVIG is unknown. To our knowledge, there are no published studies examining the effects of IVIG in this patient population. In order to proceed with a definitive clinical trial, we must establish “proof of principle”. As the risks to the subjects of this protocol are low, i.e. phlebotomy and administration of IVIG, there is a favorable risk/benefit ratio considering the importance of performing research in this area.

11. Alternatives

The alternative for patients with severe HGG to participating in this study are to not participate. As HGG is only a recently-recognized phenomenon, there is no current standard of care for this complication in solid organ transplant recipients. It is not routine to monitor or treat severe HGG in most transplant centers in the country.
